# Supplementary material for: CRISPR-Cas9/Cas12a systems for efficient genome editing and large genomic fragment deletions in Aspergillus niger
Source: Front Bioeng Biotechnol. 2024 Oct 16;12:1452496. doi: 10.3389/fbioe.2024.1452496 (PMC11521959; doi:10.3389/fbioe.2024.1452496)
Supplement: Supplementary file 3 [file DataSheet1.docx]

**Supplementary Material**

**CRISPR-Cas9/Cas12a Systems for efficient genome editing and large genomic fragment deletions in *Aspergillus niger***

Guoliang Yuan ^1,2,^*, Shuang Deng ^1,2^, Jeffrey J. Czajka ^1,2^, Ziyu Dai ^1,2^, Beth A. Hofstad ^1,2^, Joonhoon Kim ^1,2^ and Kyle R. Pomraning ^1,2,^*

^1^Energy and Environment Directorate, Pacific Northwest National Laboratory, Richland, WA, United States

^2^US Department of Energy Agile BioFoundry, Emeryville, CA, United States

*Corresponding authors: Guoliang Yuan (guoliang.yuan@pnnl.gov); Kyle R. Pomraning ([kyle.pomraning@pnnl.gov](mailto:kyle.pomraning@pnnl.gov))


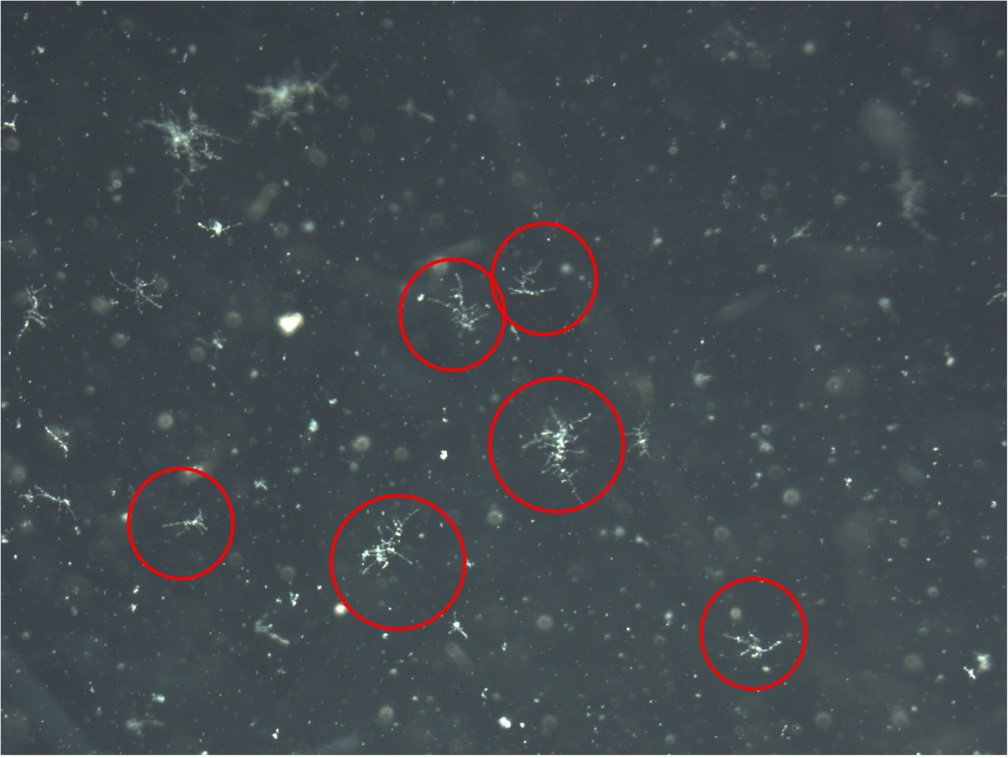


**Supplementary Figure S1. Single colonies can be identified under a dissection microscope (Leica MZ16) 19–24 hours post-transformation.**

The red circles highlight the individual colonies.


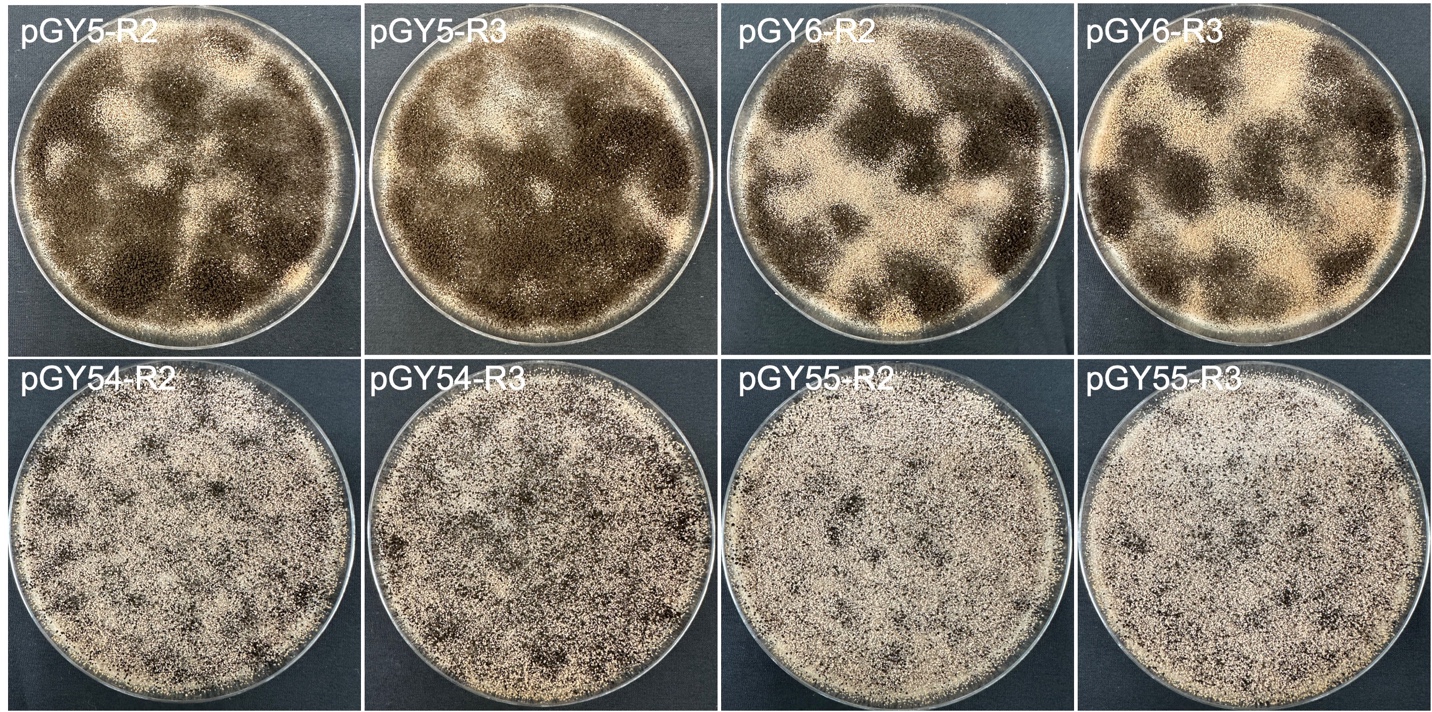


**Supplementary Figure S2. Phenotypic effects of *albA* mutations induced by Cas9 and Cas12a systems (Replicate 2 and 3).**

R stands for a biological replicate.


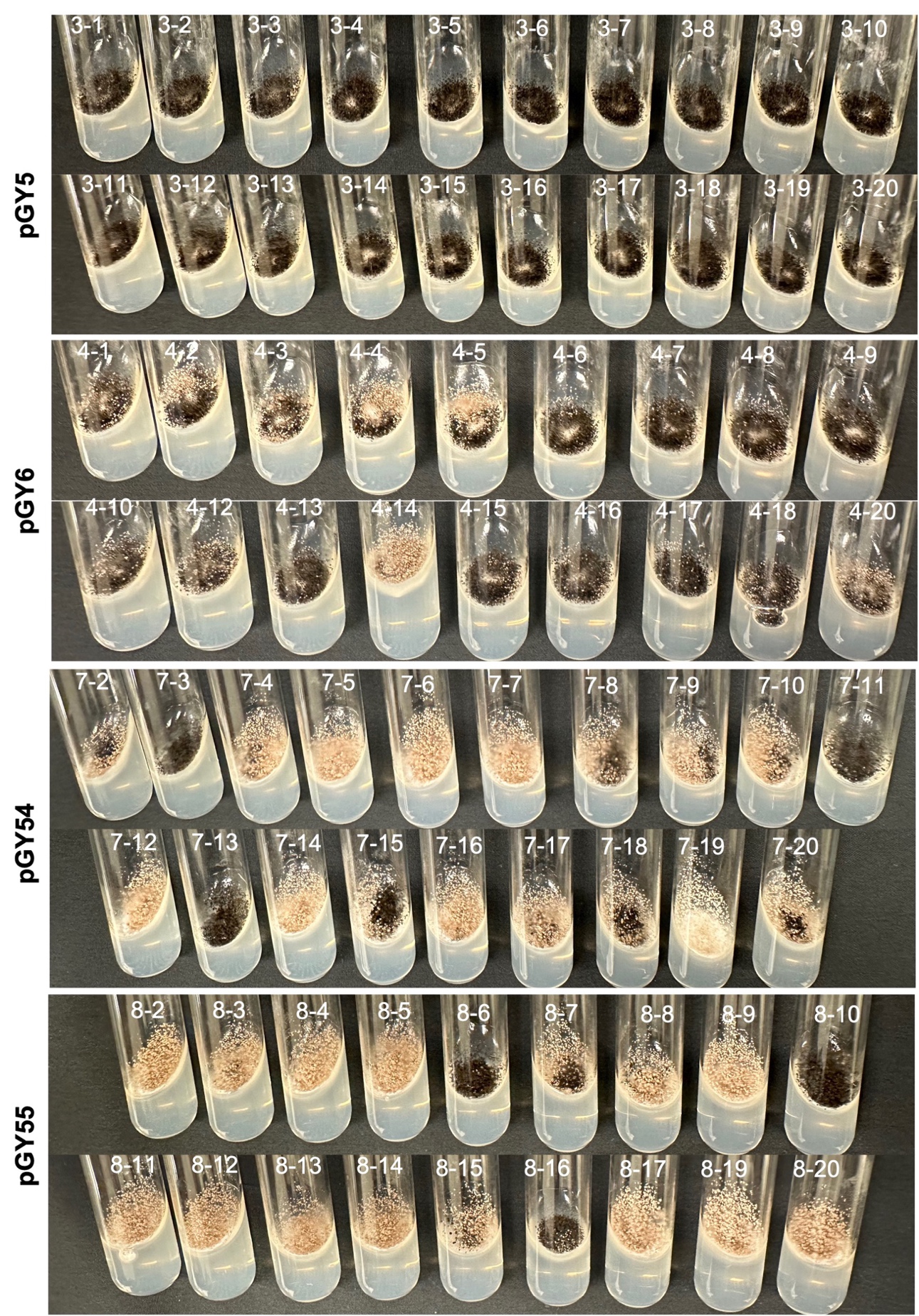


**Supplementary Figure S3. Phenotypic characteristics of transformants targeting the *albA* gene after single colony isolation on an minimal medium agar slant.**

**
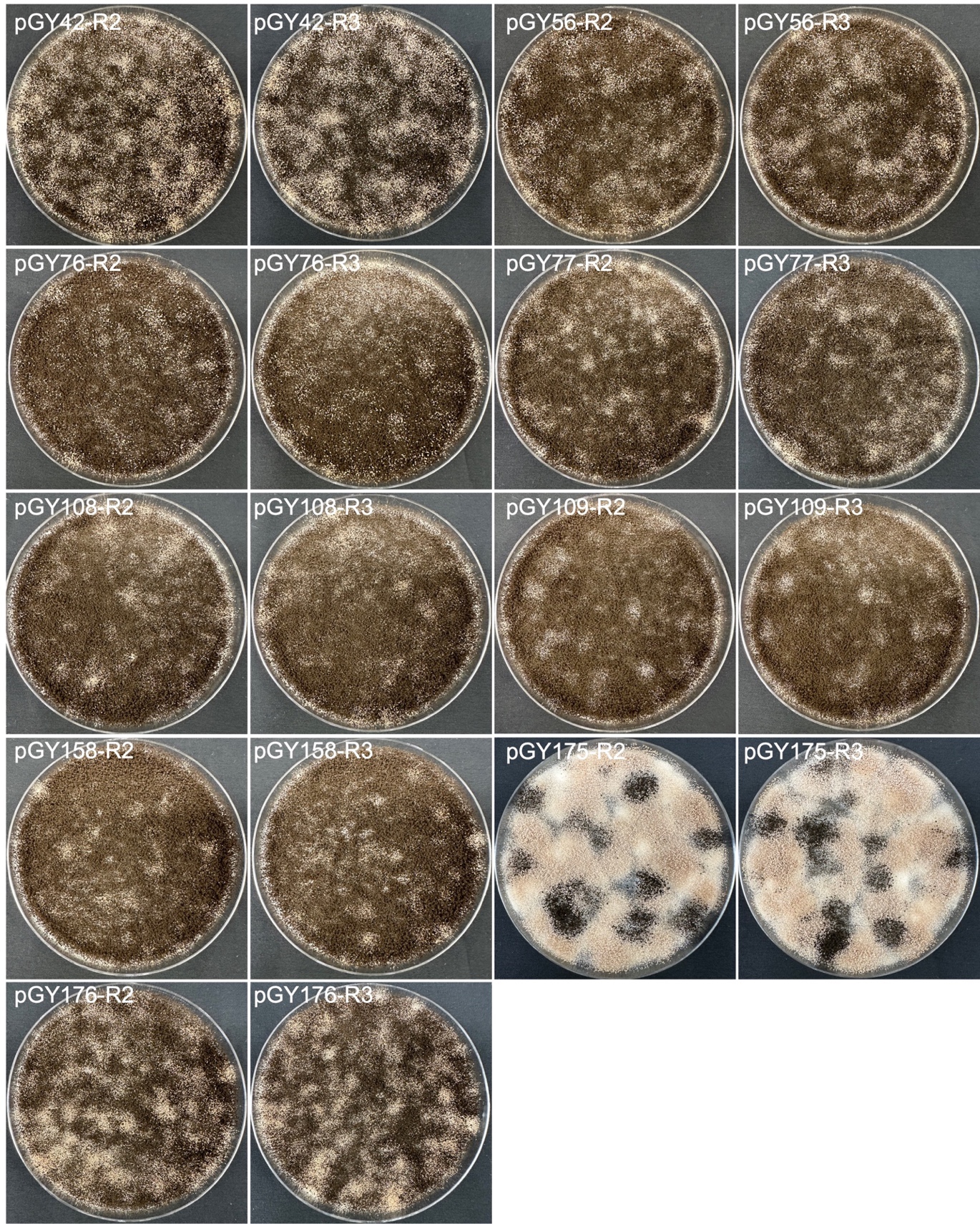
**

**Supplementary Figure S4. Phenotypic effects of large chromosome fragment deletion induced by CRISPR-Cas9 and Cas12a systems (Replicate 2 and 3).**

R stands for a biological replicate.


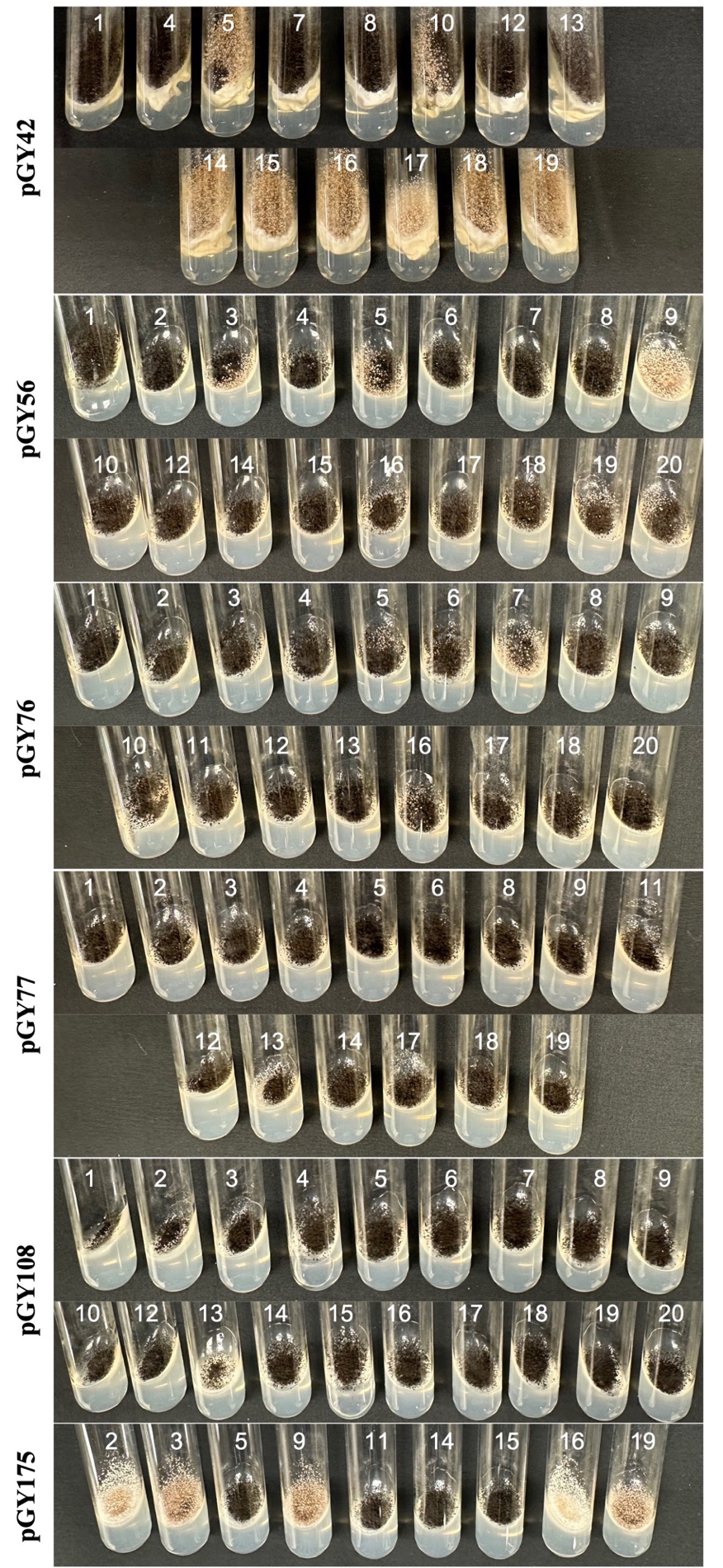


**Supplementary Figure S5. Phenotypic characteristics of selected transformants with large fragment deletions after isolating single colonies on an agar slant.**
